# Usher syndrome: clinical features, molecular genetics and advancing therapeutics

**DOI:** 10.1177/2515841420952194

**Published:** 2020-09-17

**Authors:** Maria Toms, Waheeda Pagarkar, Mariya Moosajee

**Affiliations:** UCL Institute of Ophthalmology, London, UK; The Francis Crick Institute, London, UK; Great Ormond Street Hospital for Children NHS Foundation Trust, London, UK; University College London Hospitals NHS Foundation Trust, London, UK; Development, Ageing and Disease, UCL Institute of Ophthalmology, 11-43 Bath Street, London EC1V 9EL, UK; The Francis Crick Institute, London, UK; Great Ormond Street Hospital for Children NHS Foundation Trust, London, UK; Moorfields Eye Hospital NHS Foundation Trust, London, UK

**Keywords:** gene therapy, inherited retinal disease, inner ear, photoreceptor, retina, retinitis pigmentosa, sensorineural hearing loss, sensory hair cell, Usher syndrome

## Abstract

Usher syndrome has three subtypes, each being clinically and genetically heterogeneous characterised by sensorineural hearing loss and retinitis pigmentosa (RP), with or without vestibular dysfunction. It is the most common cause of deaf–blindness worldwide with a prevalence of between 4 and 17 in 100 000. To date, 10 causative genes have been identified for Usher syndrome, with *MYO7A* accounting for >50% of type 1 and *USH2A* contributing to approximately 80% of type 2 Usher syndrome. Variants in these genes can also cause non-syndromic RP and deafness. Genotype–phenotype correlations have been described for several of the Usher genes. Hearing loss is managed with hearing aids and cochlear implants, which has made a significant improvement in quality of life for patients. While there is currently no available approved treatment for the RP, various therapeutic strategies are in development or in clinical trials for Usher syndrome, including gene replacement, gene editing, antisense oligonucleotides and small molecule drugs.

## Introduction

Usher syndrome encompasses a group of inherited disorders characterised by dual sensory impairment of the auditory and visual systems, with a variable presentation of vestibular dysfunction in a proportion of cases. It is the most common cause of combined sight and hearing loss, accounting for more than half of deaf–blindness cases.^1,2^ It has an estimated prevalence of between 4 and 17 in 100 000 people worldwide.^2,3^ Furthermore, it has been estimated to represent 5% of all congenital deafness and 18% of all retinitis pigmentosa (RP) cases.^2,4^ Usher syndrome is both clinically and genetically heterogeneous and is divided into three distinct clinical subtypes, associated with a number of genetic loci. The Usher genes encode a variety of proteins that are expressed in the inner ear and retina where they perform essential functions in sensory hair cell development and function, and photoreceptor maintenance. While many promising treatments are under investigation, there is no approved treatment for this disease to date.

## Clinical characteristics

Usher syndrome involves a combination of bilateral sensorineural hearing loss with progressive retinal degeneration in the form of RP. It is categorised into three major clinical subtypes according to severity and onset of hearing loss and whether vestibular dysfunction is present^5^ (Table 1). However, there is clinical variability within each Usher subtype, with overlapping and atypical presentations described.

**Table 1.** Clinical features and genes associated with Usher syndrome types 1, 2 and 3.

Usher subtype	Causative genes	Sensorineural hearing loss	Retinitis pigmentosa	Vestibular function
Usher 1	*MYO7A, USH1C, CDH23, PCDH15, USH1G, CIB2*	Congenital, severe to profound	Prepubertal onset; average age of diagnosis in second decade; legal blindness in fourth decade	Vestibular hypofunction; motor development may be delayed; infants typically do not walk before 18 months of age
Usher 2	*USH2A, ADGRV1, WHRN*	Congenital, moderate to severe; high frequencies most affected	Onset in second decade; average age of diagnosis in third decade; legal blindness in sixth decade.	Normal vestibular function
Usher 3	*CLRN1*	Post-lingual onset, progressive, variable	Variable onset, typically in second decade	Variable; vestibular abnormalities in ~50% of patients, usually mild

Source: Data included from previous studies.^6–10^

**Figure 1.**
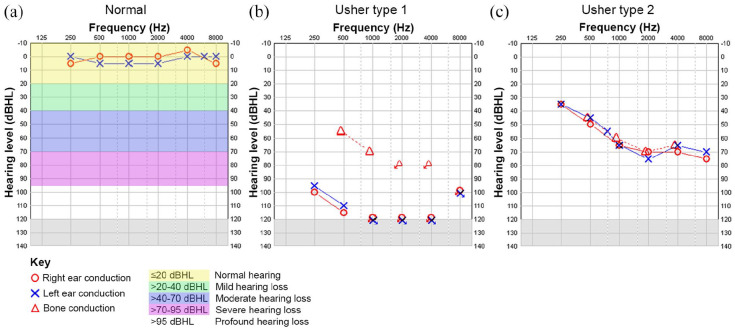
Audiograms of Usher syndrome type 1 and 2 patients. (a) Normal audiogram from a non-Usher individual. (b) Audiogram of a typical patient with Usher syndrome type 1 due to *MYO7A* mutation (homozygous c.4254del p.Asp1419fs) showing bilateral severe to profound sensorineural hearing loss (hearing loss in audiogram >95 dBHL). (c) Typical high frequency mild to severe sloping audiogram of an Usher syndrome type 2 patient (hearing loss in audiogram is 35–75 dBHL).

**Figure 2.**
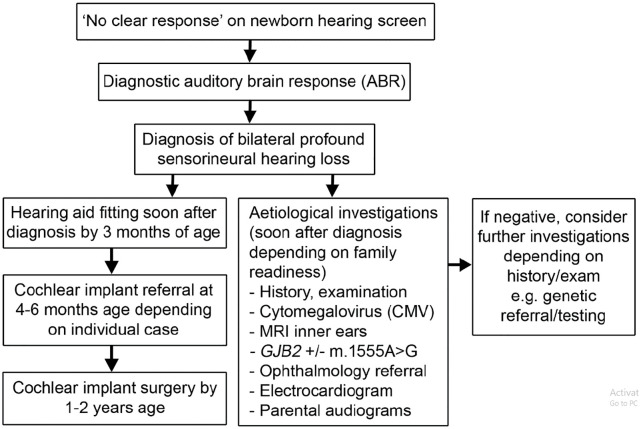
Flowchart for investigation and treatment of bilateral profound sensorineural hearing loss. Source: Adapted from the British Association of Audiovestibular Physicians (BAAP) guidelines.^12^

Usher syndrome type 1 (Usher 1) is the most severe subtype in which patients exhibit severe to profound bilateral congenital sensorineural hearing loss (Figure 1), most frequently non-progressive, with vestibular areflexia. It accounts for approximately 25–44% of all Usher syndrome cases.^11^ Infants are detected through the newborn hearing screen (Figure 2), and where not undertaken/available, the diagnosis is often suspected in infancy. Vestibular areflexia is reflected in delayed motor development and children usually do not walk independently before the age of 18 months. When older, they compensate for their vestibular areflexia using vision, until the onset of RP, although they often have higher accidental falls and difficulty in performing activities, which require balance, for example, riding a bicycle. Due to the profound nature of deafness, children with Usher 1 derive limited or no benefit from hearing aids and most patients with Usher 1 would be sign language users if the hearing loss is not treated effectively. Timely use of cochlear implants can achieve oral communication and open set speech perception. An earlier age of implantation is correlated with improved outcome.^13,14^ The standard approach is to offer bilateral cochlear implants to Usher 1 patients within the first 2 years of life.

Usher syndrome type 2 (Usher 2) is the most common form of the disorder, representing over half of all cases.^11^ The sensorineural hearing loss is typically described as sloping, mild to moderate in the low frequencies and severe to profound in the high frequencies (Figure 1).^15^ Hearing loss is congenital and infants are detected through the newborn hearing screen, however, if unavailable detection can be overlooked till the end of the first decade of life due to the high frequency configuration and degree of hearing loss.^6^ Although thought to be non-progressive, there is evidence to indicate progression of hearing loss over the years, particularly in Usher type 2A.^16–18^ Children derive benefit from conventional hearing aids and often have close to normal speech acquisition. However, with progression of hearing loss, cochlear implants are indicated. Up to 10% of Usher type 2A patients had cochlear implants (mean age: 59 years),^19^ which increased speech intelligibility, quality of life and communication, with similar outcomes to a control group of adults with post-lingual hearing loss. Vestibular function is intact in Usher 2 patients and reflected in normal motor milestones. However, one study found vestibular abnormalities in four out of five genetically confirmed Usher 2 patients.^20^ Episodes of vertigo were reported by patients although clinical balance was normal, and the authors suggested that subclinical changes in the vestibular system should be looked for.

Usher syndrome type 3 (Usher 3) is rare in most populations, accounting for approximately 2–4% of all cases, although it is particularly prevalent in Finland^21^ and among Ashkenazi Jewish people.^22^ The audiovestibular features are the most variable of the Usher subtypes. Hearing loss is of post-lingual onset and usually detected in the first decade of life, although onset can be delayed until adult life. It is typically of a progressive nature, with audiograms showing high frequencies more affected or a U-shaped configuration. Vestibular abnormalities are present in approximately half of patients, although most report a normal age of independent walking.^7^ Hearing aids are of benefit early in the course of disease, but cochlear implants may be required with progressive hearing loss. Improved hearing and subjective benefit were demonstrated in Usher 3 patients with implants (mean age at implantation: 41 years) comparable with that observed in implanted patients without visual impairment.^23^

In all types of Usher syndrome, audiological findings are detected/present prior to ophthalmological signs and symptoms. Children and adults with Usher syndrome do not have dysmorphism, and the disorder is commonly mistaken for a non-syndromic isolated sensorineural hearing loss. They will often have the initial aetiologic investigations for hearing loss, which include cytomegalovirus (CMV) testing and magnetic resonance imaging (MRI) scan, genetic testing for *GJB2* and mitochondrial m.1555A > G mutations and electrocardiography (ECG) in some patients, which is expected to be normal.^12,24^ Initial investigations for bilateral sensorineural hearing loss also include ophthalmological assessment, which in the early years can be normal depending on the Usher subtype. Diagnosis of Usher 1 should be suspected in any infant with bilateral profound sensorineural hearing loss and delayed motor milestones, even if the initial ophthalmology screen is normal. Usher 2 should be considered in patients with typical sloping configuration of hearing loss. Diagnosis of Usher 2 or 3 is made after visual symptoms or signs are detected through routine examination or electroretinography (ERG). A meta-analysis of next-generation sequencing (NGS) data in the United States indicates that 7.5% of patients with seemingly ‘isolated deafness’ have mutations in the Usher genes and may be at high risk of developing RP.^25^ With the availability of NGS, Usher genes are included in the ‘deafness’ and ‘retinal’ gene panels to help with early genetic diagnosis.

Audiological rehabilitation in all forms of Usher syndrome is started soon after diagnosis by fitting of bilateral hearing aids. Hearing aid fitting in patients with Usher syndrome may need special considerations, especially with visual impairment.^19^ The onset of RP has a significant impact on communication, as patients will have difficulty in lip reading and understanding gesture and sign, and on balance as visual compensation is compromised. Patients with Usher syndrome and bilateral vestibular areflexia should be advised about the risk of disorientation and potential drowning with underwater swimming, due to poor availability of visual and proprioceptive inputs.^26,27^

**Figure 3.**
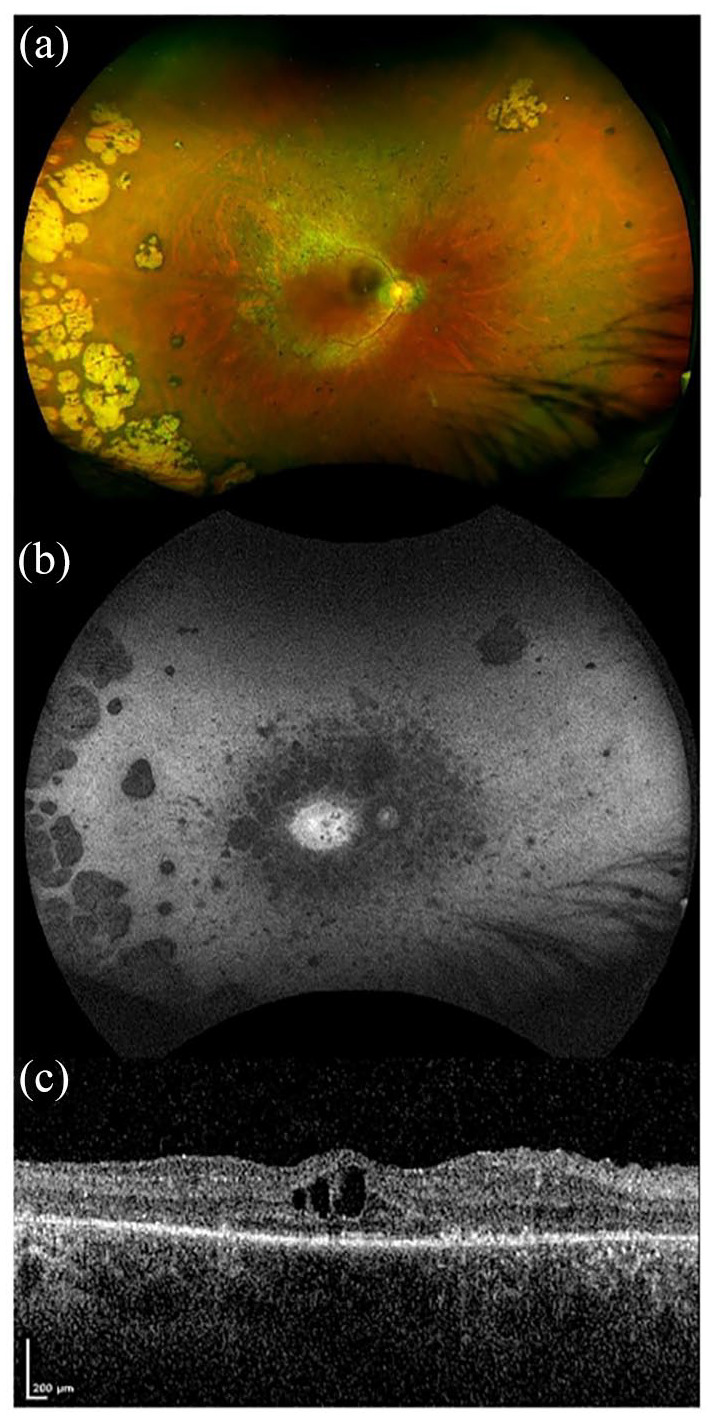
Retinal imaging of a patient with *MYO7A*-related Usher syndrome type 1. Images taken from a 34-year-old male with homozygous nonsense variants in *MYO7A*; c.2914C > T, p.(Arg972*). Best corrected LogMAR visual acuity was 0.24 in the right eye and 0.28 in the left eye. (a) Widefield colour imaging of the right fundus showing patchy RPE atrophy along the arcades with bone spicule pigmentation in the mid-periphery and peripapillary atrophy with arteriolar attenuation. In the temporal periphery, extensive chorioretinal atrophic patches are noted in this patient. (b) Widefield autofluorescence imaging of the right fundus showing dense hypoautofluorescence corresponding to RPE atrophy around the arcades extending into the mid-periphery. A ring of hyperautofluorescence is seen at the macula with speckled loss centrally. (c) Spectral-domain optical coherence tomography (SD-OCT) of the right eye showing loss of retinal lamination, cystoid macular oedema with intraretinal cystic changes and extensive loss of the ellipsoid zone.

**Figure 4.**
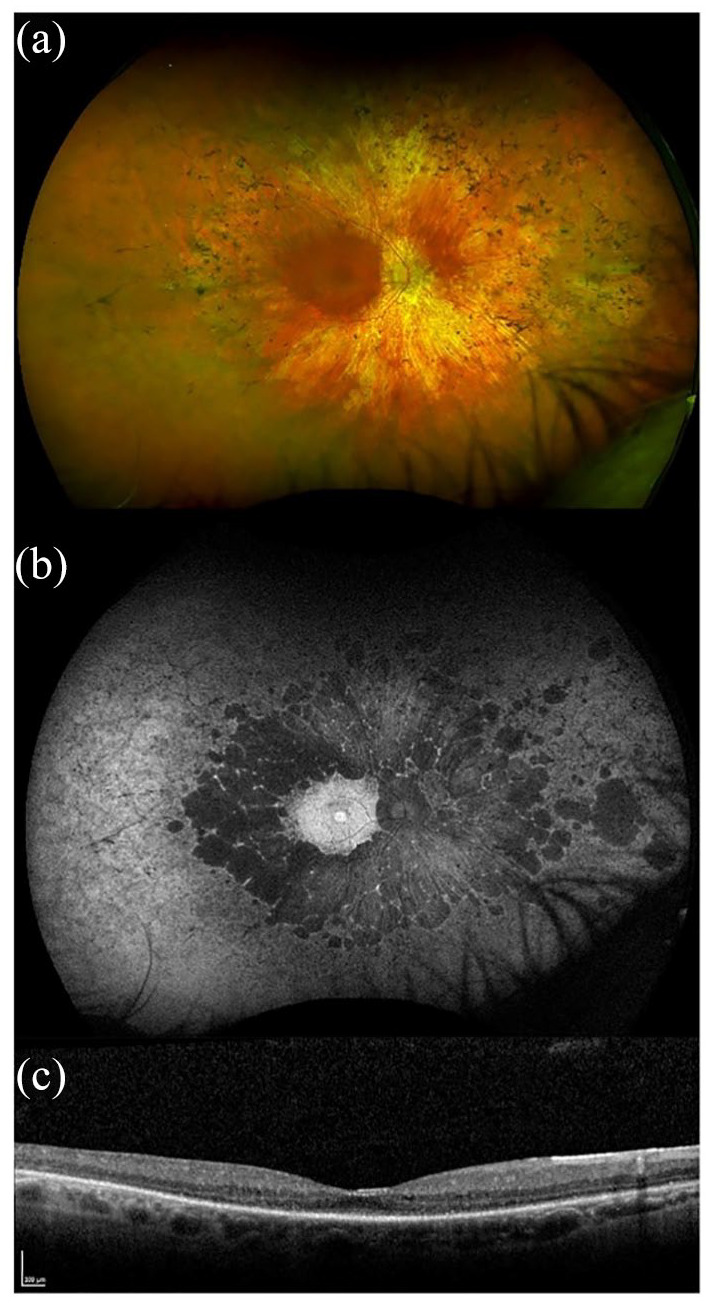
Retinal imaging of a patient with *USH2A*-related Usher syndrome type 2. Images taken from a 58-year-old male with compound heterozygous variants in *USH2A*; c.2299delG, p.(Glu767Serfs*21) and c.100C > T, p.(Arg34*). Best corrected LogMAR visual acuity was 0.50 in the right eye and 0.30 in the left eye. (a) Widefield colour imaging of the right fundus showing scattered bone spicule pigmentation in the mid-periphery and areas of depigmentation with RPE atrophy. Preserved retinal island at the macula, arteriolar attenuation and a waxy pale disc. (b) Widefield autofluorescence imaging of the right fundus showing hyperfluorescence signal at the fovea with dense scalloped hypoautofluorescence around the macula, arcades and extending past the mid-periphery corresponding with RPE atrophy. (c) Spectral-domain optical coherence tomography (SD-OCT) of the right eye showing retinal thinning and a small residual ellipsoid zone.

RP develops in all three Usher subtypes but with variable onset; Usher 1 is most commonly pre-adolescent, with Usher 2 within the first two decades of life, and Usher 3 patients typically being post-pubertal.^8,28^ Visual prognosis also differs between the clinical types; Usher 1 patients generally show a more severe visual decline with age, reaching legal blindness on average 15 years earlier than patients with Usher 2.^6,9^ Typically, the first presenting symptom is night blindness (nyctalopia) with progressive visual field loss beginning in the mid-periphery caused by rod photoreceptor degeneration. It eventually progresses to involve cone photoreceptors, resulting in central and colour vision loss. Patients are often registered severely sight impaired but there can be significant intra- and interfamilial phenotypic variability. Fundus features include variable amounts of bone spicule pigmentation, retinal pigment epithelium (RPE) atrophy or depigmentation, retinal arteriolar attenuation and optic disc pallor (Figures 3(a) and 4(a)). A significant proportion of Usher patients may also develop cataracts and/or cystoid macular oedema.^29^

Retinal imaging using fundus autofluorescence shows a ring of hyperautofluorescence in the macula (Figures 3(b) and 4(b)), and spectral domain optical coherence tomography (SD-OCT) reveals loss of the outer retinal structure (Figures 3(c) and 4(c)), sparing the fovea until late in disease ± cystoid macular oedema. A prospective study using optical coherence tomography angiography (OCTA) of patients with *MYO7A* and *USH2A* mutations showed reduced vessel density in the retinal circulation with changes in the superficial capillary plexus (SCP) and deep capillary plexus (DCP) in all patients compared with healthy age-matched controls.^30^ However, peripheral defects were detected in the choriocapillaris (CC) earlier and more severely in *MYO7A* patients compared with the *USH2A* group. It was suggested that this is because the *MYO7A* protein (myosin VIIa) is mainly expressed in the RPE, thus affecting the CC directly. In the same study, patients were tested for macular sensitivity (MS) using microperimetry with the Macular Integrity Assessment, and this detected decreased mesopic mean MS in all patients, especially in the periphery. Patients with *MYO7A* mutations had a slightly lower mean MS than patients with *USH2A* mutations; however, the differences were found not to be statistically significant (*p* = 0.66). Static and dynamic perimetry detects mid-peripheral visual field loss with progression to residual small central islands, with a small temporal peripheral field preservation in the most advanced stages of the disease. ERG measurement can show reduction and delay of amplitudes in the early stages of the disease, and is a useful test to perform in an infant born with profound deafness to determine the likelihood of underlying Usher 1, even before visual dysfunction is otherwise noted. Later, the full-field ERG is often non-recordable.

Dual sensory clinics are now being established to improve the clinical pathways and experience of children with hearing and sight impairment. These clinics will provide access to the relevant multidisciplinary in one visit, hence reducing stress and the burden associated with numerous, separate medical appointments. Children with Usher syndrome have been reported to develop mental and behavioural disorders, including autism, conduct disorder, schizophrenia and learning difficulty. This could be multifactorial, due to sensory deprivation, stress, difficulty in diagnosis and a possible unproven genetic association.^31,32^ Dual sensory clinics will promote faster accurate diagnosis through more extensive genetic testing and detection of visual symptoms and mental health issues at an earlier stage.

## Genetics of Usher syndrome

### Usher genes

All Usher syndrome types are inherited in an autosomal recessive manner. To date, at least 10 causative genes have been identified for the disease, which include six Usher 1 genes, three Usher 2 genes and one Usher 3 gene. Historically, traditional Sanger sequencing of all Usher gene exons was found to provide a genetic diagnosis for more than 80% of Usher families,^33,34^ but this is time-consuming and costly, particularly for large patient cohorts. Microarray-based testing provided ~33% detection for Usher patients but can only screen for known mutations.^35,36^ NGS, including whole exome and genome sequencing, is now the method of choice with high efficiency offering the advantage of detecting a range of known and novel mutations, including large genomic DNA rearrangements. Targeted exome gene panel testing can reach diagnostic rates of around 70–80% in Usher families.^29,37–39^

Currently, there are nine loci (USH1B-J) known to be involved in Usher 1. The genes identified for six of these loci are as follows: *MYO7A* (USH1B),^40^ *USH1C* (USH1C),^41,42^ *CDH23* (USH1D),^43^ *PCDH15* (USH1F),^44^ *USH1G* (USH1G),^45^ and *CIB2* (USH1J).^46^ Of these genes, *MYO7A* is the most frequent cause of Usher 1, accounting for more than half of cases.^34^ The USH1E, USH1H and USH1K loci have been mapped to chromosomes 21q21, 15q22–23 and 10p11.21–q21.1, respectively,^47–49^ but the genes are yet to be identified. It is worth noting that *CIB2* bi-allelic loss of function variants has been reported in patients with non-syndromic recessive hearing loss (DFNB48) but with no retinal symptoms.^50^

Three genes underlying Usher 2 have been identified as *USH2A* (USH2A),^51^ *ADGRV1* (USH2C)^52^ and *WHRN* (USH2D).^53^ *USH2A* mutations are the most common cause of Usher syndrome, accounting for around 80% of Usher 2 cases.^34^ In addition, *PDZ domain-containing 7* (*PDZD7*) has been reported to act as a disease modifier and contributor to a digenic form of Usher 2.^54^

*CLRN1* (or *USH3A*) is the only gene currently confirmed to cause Usher 3,^55,56^ with two prevalent mutations, p.(Tyr176*) and p.*Asn48Lys), accounting for most cases in Finnish and Ashkenazi Jewish patients, respectively.^22,57^ A homozygous missense variant in *histidyl-tRNA synthetase* (*HARS*) has also been reported in two patients with a phenotype compatible with Usher 3 (sometimes referred to as USH3B).^58^

The Usher genes encode a number of structurally and functionally distinct proteins; these include an actin-binding motor protein (myosin VIIA, USH1B^40^), scaffolding proteins (harmonin, USH1C;^41,42^ sans, USH1G;^45^ whirlin, USH2D^53^), cell adhesion/transmembrane proteins (cadherin 23, USH1D;^43^ protocadherin 15, USH1F;^44^ usherin, USH2A;^51,59^ clarin-1, USH3A^56^), an adhesion G-coupled receptor (ADGRV1, USH2C^52^) and a calcium- and integrin-binding protein (CIB2, USH1J^46^). Most are expressed as multiple splice and protein variants in a range of tissues,^59–64^ but all of the Usher proteins are present in the inner ear and retina where most have been found to interact and form complexes that localise to subcellular locations in the ciliated sensory neurons, that is, inner ear hair cells and retinal photoreceptors.^65,66^ Myosin VIIA is also an essential RPE protein,^67–69^ and evidence suggests that clarin-1 is restricted to the retinal Müller glia.^70^ Various studies have indicated the involvement of Usher proteins in a range of processes, including cohesion, mechanotransduction, synaptic maturation, and protein and organelle transport.

### Genotype–phenotype correlations

The Usher genes show vast clinical heterogeneity and different mutations in most Usher genes have been linked to non-syndromic cases of autosomal recessive RP, or autosomal dominant or recessive sensorineural hearing loss (annotated as DFNA or DFNB).^46,53,71–80^ These include mutations in *MYO7A*, which have been associated with dominant and recessive non-syndromic hearing loss (DFNA11 [OMIM #601317] and DFNB2 [OMIM #600060], respectively).^81,82^ It has been suggested that mutations that allow some residual motor protein function, for example, in-frame deletion c.5146_5148delGAG p.(Glu1716del), cause the milder non-syndromic phenotypes, whereas mutations associated with Usher 1, for example, c.1309G > A p.(Asp437Asn), have a more severe effect on protein function.^83^ A separate study of 33 USH1B patients proposed that null *MYO7A* alleles, that is, those with stop mutations within the motor domain coding region such as c.999T > C p.(Tyr333*), may cause milder visual dysfunction than missense variants owing to a lack of mutant protein contributing to disease pathology.^10^ Significant correlations have not been reported in other patient populations with *MYO7A* mutations.^84^ Mutations of *MYO7A* reported to cause DFNB2 are comparable with those causing Usher 1, leading to question whether this phenotype results from missed RP or whether there may be modifying factors which influence the phenotype.^85^ *MYO7A* mutations have also been reported to cause a phenotype of unilateral auditory neuropathy in a Chinese family with Usher 1^86^ and an Usher 2 phenotype,^87^ expanding its phenotypic spectrum.

Among the other Usher 1 genes, there is evidence of a genotype–phenotype correlation in both *CDH23* and *PCDH15*; missense variants are primarily associated with non-syndromic deafness (DFNB12 [OMIM #601386] or DFNB23 [OMIM #609533]) or more subtle RP symptoms, whereas frameshift, nonsense and splice site mutations cause Usher 1.^72,74,88–90^ However, missense mutations in *CDH23* can also cause milder or ‘atypical’ Usher 1, and genotype–phenotype correlations are not always predictable.^88^ Pathogenic *USH1C* variants, including a leaky splice site mutation IVS12 + 5G > C,^73^ have also been shown to cause recessive non-syndromic hearing loss (DFNB18 [OMIM #602092]), which has been proposed to be related to the less deleterious effect of the variants being located within alternatively spliced exons.^73,91^ A family with non-syndromic sensorineural hearing loss caused by compound heterozygous missense and frameshift mutations in *USH1G* has been described;^78^ missense mutations in *USH1G* that are expected to result in residual protein function have been reported in Usher 1 families, thus expanding the phenotypic heterogeneity of Usher 1G disease.

*USH2A* has a diverse mutation spectrum, which includes nonsense, frameshift, missense and splice-affecting mutations, as well as deletions and duplications. The most common mutation found in *USH2A* is a single base pair (bp) deletion in exon 13, c.2299delG p.(Glu767Serfs*21),^34,92^ which has been shown to be associated with exon splicing.^93^ This variant is predicted to produce a severely truncated protein and/or be subject to nonsense-mediated decay; however, transcript analysis showed that it caused skipping of exon 13 or exons 12 and 13.^93^ Mutations of *USH2A* are associated with up to 23% of non-syndromic RP cases,^94^ and specific mutant alleles are more frequent among such patients and families, the most common being missense variant c.2276G > T p.(Cys759Phe).^95,96^ Unusually, one patient with compound heterozygous *USH2A* mutations, c.1036A > C p.(Asn346His) and c.13316C > T p.(Thr4439Ile), was reported to have non-syndromic hearing loss, while their sibling harboured the same mutations and was diagnosed with typical Usher 2.^97^

Several *USH2A* phenotype-genotype studies have been carried out to date. A survey conducted by Lenassi and colleagues of patients with *USH2A*-associated RP reported several ‘retinal disease-specific’ alleles that were rarely found in Usher 2 families, mostly missense variants that were likely to be less deleterious, while the Usher-associated variants mostly included those that were predicted to produce no viable protein (e.g. those causing premature truncation).^96^ They proposed an allelic hierarchy model in which the presence of at least one retinal disease-specific allele in a patient with *USH2A*-related retinopathy resulted in the preservation of hearing. While this has not been supported by subsequent studies,^6,98,99^ the same analysis on a different cohort^100^ combined with two large external cohorts^98,101^ found that the allelic hierarchy model was valid in 86% of individuals with non-syndromic *USH2A*-RP.^100^ In addition, it has been reported that Usher 2 patients with one copy of the p.(Cys759Phe) allele showed a later onset of RP and milder hearing loss compared with the general Usher 2 population,^6^ and the presence of the p.(Cys759Phe) variant in a homozygous state or in combination with other *USH2A* missense mutations has been associated with isolated RP or RP with late onset hearing loss.^99^ In contrast, the p.(Glu767Serfs*21) variant results in a more rapid deterioration and severe hearing threshold, heralding the need for careful audiological monitoring and consideration of cochlear implants.^100^ In general, severe hearing impairment has been associated with truncating variants in *USH2A.*^6,99,101,102^ Further investigations into *USH2A* genotype–phenotype correlations have reported that the presence of two truncating mutations, or two missense variants in the N-terminal laminin domain of the gene, were associated with Usher 2 patients and not those with non-syndromic RP,^98,103^ and the presence of at least one truncating mutation was related to earlier visual decline regardless of the phenotype.^98,99^

*WHRN* is an additional Usher gene that causes non-syndromic deafness, which is related to the mutation location affecting the two predominantly expressed variants (long and short): N-terminal mutations that affect the long isoform are found in USH2D patients,^53,104^ whereas mutations in the C-terminal region manifest as DFNB31.^105,106^

## Development of therapies

### Preclinical studies

While there is currently no available cure, there are numerous therapeutic strategies under development for Usher-related RP and other inherited retinal diseases (IRDs): these include gene replacement, gene editing, nonsense suppression and antisense oligonucleotide (ASO)–based approaches (Table 2). The eye is an attractive organ for therapeutic applications due to its accessibility and immune privilege, while the natural history of the disease with preserved cone photoreceptors at the fovea until a later stage provides an ideal window for intervention. Most therapeutic studies for Usher syndrome have been performed using patient-derived cells (typically fibroblasts) or mutant mice, of which there are many, with at least one existing for each causative gene.^65,66^ Most Usher mice display sensorineural hearing loss and vestibular phenotype reminiscent of their human counterparts, with only a limited number showing progressive retinal degeneration. Despite this, they have still aided in the assessment of potential treatments.

**Table 2.** Treatment approaches for Usher syndrome.

Gene	Treatment type	Method	Model(s) tested	Clinical trials	References
Usher 1					
*MYO7A*	Gene replacement	Subretinal injection of AAV vectors	*Myo7a*^–/–^ mice		Previous studies^107–110^
		Subretinal injection of dual AAV vectors	*Myo7a*^–/–^ mice		Lopes and colleagues,^108^ Trapani and colleagues,^109^ Colella and colleagues^111^
		Subretinal injection of lentiviral vectors	*Myo7a*^–/–^ mice	NCT01505062, NCT02065011	Hashimoto and colleagues,^112^ Zallocchi and colleagues^113^
*USH1C*	Gene replacement	Round window membrane injection of AAV vectors	*Ush1c* knock-in (c.216G > A) mouse		Pan and colleagues^114^
	Gene editing	Transfection of zinc finger nucleases and HDR template plasmid	HEK293 cell line transfected with *Ush1c* c.91C > T p.(Arg31*)		Overlack and colleagues^115^
	Nonsense suppression	Incubation with NB30, NB54 and PTC124	HEK293 cell line and mouse retinas transfected with *Ush1c* c.91C > T p.(Arg31*),		Goldmann and colleagues^116,117^
	ASO	Peritoneal injection or transuterine injection into the amniotic cavity or inner ear of ASOs designed to correct defective pre-mRNA splicing	*Ush1c* knock-in (c.216G > A) mice		Previous studies^118–120^
*PCDH15*	Nonsense suppression	Incubation with gentamicin, paromomycin, NB30, NB54	COS-7 cell line transfected with mutant *PCDH15* constructs		Nudelman and colleagues^121–123^
*USH1G*	Gene replacement	Round window membrane injection of AAV vectors	*Ush1g*^–/–^ mice		Emptoz and colleagues^124^
Usher 2					
*USH2A*	Gene editing	Transfection of CRISPR/Cas9 components and HDR template	Patient fibroblasts and iPSCs with compound heterozygous *USH2A* c.2299delG p.(Glu767Serfs*21) and c.2276G > T p.(Cys759Phe) or homozygous p.(Glu767Serfs*21)		Fuster-Garcia and colleagues,^125^ Sanjurjo-Soriano and colleagues^126^
	Nonsense suppression	Incubation with PTC124	HEK293 cell line transfected with *USH2A* c.11864G > A p.(Trp3955*). Patient fibroblasts with *USH2A* c.9424G > T p.(Gly3142*)		Neuhaus and colleagues,^127^ Samanta and colleagues^128^
	ASO	Transfection with ASOs designed to correct defective pre-mRNA splicing	Patient fibroblasts with *USH2A* c.7595-2144A > G and minigene splice assay		Slijkerman and colleagues^129^
		Treatment with ASO (QR-421a) designed to induce skipping of *USH2A* exon 13	Patient iPSC-derived retinal organoids with *USH2A* p.(Glu767Serfs*21), *ush2a*^rmc1^ zebrafish, wild-type macaque, wild-type mice	NCT03780257	ProQR Therapeutics^130^
*USH2D*	Gene replacement	Subretinal injection of AAV vectors	*Whirlin*^−/−^ mice		Zou and colleagues^131^
		Round window membrane injection or posterior semicircular canal injection of AAV vectors	*Whirler* mice		Isgrig and colleagues,^132^ Yasuda and colleagues^133^
Usher 3					
*CLRN1*	Gene replacement	Subretinal or intravitreal injection of AAV vectors	Wild-type mice		Dinculescu and colleagues^134^
		Round window membrane injection of AAV vectors	*Clrn*^–/–^ (KO-TgAC1)		Previous studies^135–137^
		Round window membrane injection of AAV vectors	*Clrn1*^ex4–/–^, *Clrn1*^ex4fl/f^ *Myo15-Cre*^+/–^ mice		Dulon and colleagues^136^
		Round window membrane injection of AAV vectors	*Clrn*^–/–^ mice, wild-type rats and macaque		Isgrig and colleagues^132^
	Small molecule drug	Peritoneal injection of BioFocus 844, identified as stabilising CLRN1^N48K^ protein	*Clrn1* ^N48K/N48K^		Alagramam and colleagues^138^
Non-gene-specific					
	Cell transplant	Subretinal injection of human neural progenitor cells	*Ush2a*^–/–^ mice		Lu and colleagues^139^
		Intravitreal implantation of encapsulated cells expressing CNTF	Rodent models of retinal disease	NCT00447980, NCT01530659	Previous studies^140–143^
		Retrobulbar, subtenons, intravitreal, subretinal, intra-optic nerve and intravenous injections of BMSC		NCT01920867, NCT03011541	Weiss and colleagues^144^

AAV, adeno-associated virus; ASO, antisense oligonucleotides; BMSC, bone marrow–derived stem cells; CNTF, ciliary neurotrophic factor; HDR, homology-directed repair; HEK, human embryonic kidney; iPSC, induced pluripotent stem cell.

Gene replacement is an approach that has been shown to be effective in several Usher mouse models: adeno-associated virus (AAV) vectors have been used in *Myo7a*,^107–110^ *Whrn*^131^ and *Clrn1*,^134^ knockout mice via subretinal injection to restore expression of the wild-type Usher gene that was defective in each model. Dual overlapping AAV vectors have also been tested for *MYO7A* delivery as a potentially safer alternative for large genes with promising results, although they were not found to be as efficient.^108,109,111^ Alternatively, delivery of functional *MYO7A* to the USH1B mouse model retina via lentiviral-based vectors with larger carrying capacities (9 *versus* 4.7 kb for single AAV) proved successful,^112,113^ although it harbours the risk of insertional mutagenesis. Among the other Usher models, gene delivery using AAV vectors has also produced significant improvements in auditory and vestibular hair cell function in mouse models of USH1C,^114^ USH1G,^124^ USH2D,^132^ and USH3.^135–137^ This was achieved by viral injection into the inner ear through the round window membrane^114,124,135–137^ or posterior semicircular canal^132^ in postnatal neonatal mice.

The mouse inner ear is immature at birth and continues to mature postnatally. The acquisition of hearing (measured by the onset of startle response) occurs 12 days postnatally in mice, providing a window of opportunity for effective intervention with gene therapy.^145^ Comparatively, hearing in humans is fully mature at birth (onset of startle response at 19 weeks gestation). The hearing loss in Usher 1 is established at birth and it is not clear whether the hair cells in the human inner ear are a viable therapeutic target. In order to be effective, human intervention should be considered within the foetal stage, before establishment of hearing (at ~18 weeks of gestation).^145^ Hence, where therapeutic response in mice is successful when given soon after birth, it is questionable whether the same effect will be seen in patients with postnatal treatment. Overall, further studies in non-human primates will be useful in addressing some of these issues.^66^

One alternative approach to gene replacement is gene editing, which involves cutting around genetic mutations through the use of nuclease enzymes and correcting the DNA error by homologous recombination with a DNA template containing the wild-type sequence.^146^ This can be used to correct point mutations, small indels and splice site mutations, and is suitable for any gene size. Early investigations into the use of this strategy for Usher-directed treatment employed the use of two zinc finger nucleases to correct an *USH1C* point mutation (c.91C > T p.[Arg31*]) and induce full-length harmonin expression in cultured cells.^115^ In recent years, the CRISPR/Cas9 system has become highly popular for gene editing due to its efficiency and ease of use. This technique has been used for successful *in vitro* mutation repair in *USH2A* patient fibroblasts harbouring homozygous p.(Glu767Serfs*21) mutations,^125^ as well as patient-derived induced pluripotent stem cells (iPSCs) either homozygous for *USH2A* p.(Glu767Serfs*21) mutations or compound heterozygous for p.(Glu767Serfs*21) and p.(Cys759Phe).^126^ However, the efficiency of mutation correction was only 2.5% in fibroblasts^125^ and up to 3% in iPSCs,^126^ although the second study reported an 80% editing efficiency in the small number of homozygous *USH2A* clones that survived. Encouragingly, neither study reported off-target effects, which are unwanted mutations induced at DNA locations that show homology to the guide sequence. CRISPR/Cas9-based editing shows huge promise for the treatment of IRDs caused by a range of mutations; however, ensuring the absence of off-target effects and a high level of editing efficiency in retinal cells will be essential for future investigations.

Small molecule-based methods for treatment of Usher syndrome have included the use of translational read-through-inducing drugs (TRIDs), which bind to the translational machinery and are able to induce insertion of an amino acid at the site of premature stop codons, allowing read-through of nonsense mutations. These small molecule drugs include ataluren (PTC124) and designer aminoglycosides (NB compounds such as NB54). Several TRIDs have been used to suppress Usher 1-associated *PCDH15* and *USH1C* nonsense mutations *in vitro*, in cell cultures and in retinal explants.^116,117,121–123^ Furthermore, *in vivo* administration of NB54 and PTC124 was able to restore expression of full-length harmonin in mouse retinas transfected with *Ush1c* reporter constructs.^117^ For Usher 2 investigation, PTC124 was administered to a human embryonic kidney (HEK) cell model expressing a cDNA fragment of *USH2A* containing the c.11864G > A p.(Trp3955*) mutation and showed a substantial increase in *USH2A* expression compared with the control.^127^ Further studies demonstrated PTC124 efficacy in restoring *USH2A* protein expression and primary ciliogenesis capability in *USH2A* patient-derived fibroblasts with the c.9424G > T p.(Gly3142*) mutation.^128^ Overall, TRIDs show promise as a safe and effective strategy to treat a range of Usher-related nonsense mutations; however, these particular variants cause ~16% of *USH2A*-related RP and 13% of all IRD cases.^128^

An additional small molecule that has been of interest for Usher syndrome treatment, known as BioFocus 844 (BF844), was identified through cell-based high throughput screening as capable for stabilising the defective Clarin-1 protein produced by the common *CLRN1* missense variant p.(Asn48Lys).^138^ BF844 was shown to protect against progressive hearing loss when administered intraperitoneally to an Usher 3 knock-in mouse model.

A further therapeutic option is the use of ASOs, which are short synthetic modified nucleic acids that bind RNA through complementary base pairing. They can be designed to bind pre-mRNA at splice enhancer or silencer target sites, preventing or stimulating binding of the spliceosome thereby modulating pre-mRNA splicing. ASOs have been used to rescue both the hearing and vestibular defects in *Ush1c* knock-in mice, which possess a cryptic splice site mutation that results in truncated harmonin protein.^118–120^ Initially, peritoneal injection of ASOs in neonatal mice was shown to partially correct defective pre-mRNA splicing of mutant *Ush1c* transcripts; the same group delivered ASOs to *Ush1c* knock-in foetal mice *in utero* via transuterine injection into the amniotic cavity and observed partial correction of vestibular function and hearing in the mice postnatally,^119^ while most recently transuterine injection directly into the developing inner ear produced more substantial improvements in both hearing and vestibular function that sustained into adulthood.^120^ ASOs have also been used to correct a splicing defect caused by a deep intronic mutation in the *USH2A* gene (c.7595-2144A > G) which leads to insertion of a pseudoexon (PE40), in both patient-derived fibroblasts and a minigene splice assay.^129^

Overall, there are a number of promising therapeutic strategies in the development for the Usher subtypes. The use of patient-derived retinal organoids, which have already been generated for Usher syndrome caused by *USH2A* mutations,^147,148^ will further aid in the testing of novel treatments by providing the opportunity to demonstrate therapeutic potential in retinal-specific cells *in vitro*.

### Identifying outcomes

Although several treatment strategies are already under development for Usher syndrome and other IRDs, identifying metrics that display detectable changes within relatively short time periods (e.g. 1–2 years) in otherwise slowly progressive conditions will aid the assessment of therapeutic efficacy in clinical trials. This is especially important when the treatments are administered systemically, such as orally, and the untreated eye cannot be used as a control. As the hearing loss is congenital and relatively stable throughout the lifetime of Usher patients (aside from Usher 3), a number of natural history studies have focussed on the progress of the retinal disease in Usher patients; these have included longitudinal assessment of patients with *MYO7A*^9,10,84,149^ and *USH2A*^9,98,150–152^ mutations using various clinical functional and structural measures, including visual acuity, perimetry, ERG, fundus autofluorescence and OCT-derived measurements.

For Usher 2, longitudinal data from a cohort of patients carrying the common *USH2A* c.2299delG mutation were studied and rod perimetry across the visual field was highlighted as a potential clinical measure for timely investigations, predicted to show detectable change within 1.4 years.^150^ Ellipsoid zone (EZ) line width, which is the inner/outer segment boundary measured from OCT scans, was an effective surrogate measure of central visual loss and was predicted to show a detectable decrease in 2.3 years. A recent investigation into an Usher 2 patient population carrying a range of *USH2A* variants used retrospective longitudinal data to identify suitable clinical outcome metrics.^151^ Both EZ line and hyperautofluorescent outer retinal ring area showed significant reductions within the follow-up period (2–5 years); however, there was considerable variability in the population. Visual acuity was not found to be a suitable measurement due to its slow decline, consistent with previous work.^150^ Furthermore, measuring retinal thickness from OCT images was confounded by the presence of macular oedema.

Data from ongoing longitudinal natural history studies, such as the ‘Rate of Progression in *USH2A*-related Retinal Degeneration’ (RUSH2A, NCT03146078) and the ‘Multicentre Longitudinal, Observational Natural History Study to Evaluate Disease Progression in Subjects With Usher Syndrome Type 1B’ (NCT03814499), in addition to the use of artificial intelligence–based methods will further aid in identifying suitable outcome metrics for clinical trials for Usher syndrome. This will likely be tailored to the mechanism (gain of function or slowing of disease progression) and target of treatment, that is, retina-wide or central retina. If gain of function is anticipated, trials could be relatively short (between 12 and 18 months) to arrive at an estimate of potential longevity.

### Clinical trials

Owing to the success of preclinical investigations, there are several completed and ongoing clinical trials for patients with Usher-related RP. For Usher-specific gene therapy, the first clinical trial evaluated subretinal injection of a recombinant equine infectious anaemia virus (EIAV)–based lentiviral vector for delivery of *MYO7A* cDNA (UshStat) for treating patients with *MYO7A*-related Usher 1 (NCT01505062).^113^ However, this phase I/IIA trial has been terminated by the sponsor Sanofi due to review of clinical development plans and priorities. A second trial is ongoing to assess long-term safety of patients who received UshStat (NCT02065011). A further clinical trial is being prepared using dual hybrid AAV vectors to deliver *MYO7A* to the retina of USH1B patients (https://cordis.europa.eu/project/id/754848/it).^109,153^ Considering the Food and Drug Administration and European Medicines Agency approval of Spark Therapeutics Luxturna gene therapy for patients with *RPE65*-related retinal disease, gene replacement therapy has become a more likely future option for the treatment of several Usher subtypes. However, such therapies are likely to be highly costly, and conventional viral methods are not appropriate for very large genes like *USH2A* (cDNA length >15 kb).

For ASO-based treatments, there is currently a trial sponsored by ProQR for an ASO candidate (QR-421a), which has been designed to exclude the whole exon 13 in the *USH2A* mature mRNA transcript; this has been shown preclinically to result in restoration of functional usherin protein.^130^ Considering that two of the most common pathogenic *USH2A* mutations occur in exon 13,^34,92,96^ if successful this treatment would be suitable for a large proportion of patients with *USH2A*-related Usher 2 and RP. The phase I/II clinical trial is currently ongoing for intravitreal injection of QR-421a in patients with *USH2A* exon 13 variants (NCT03780257).

In addition to Usher gene-specific clinical trials, subretinal implantation of capsules containing human NT-501 cells that release ciliary neurotrophic factor (CNTF) has been trialled in patients with choroideremia and RP, including some with Usher 2 and Usher 3 (NCT00447980, NCT01530659).^140,141^ CNTF has been found to prolong photoreceptor survival in mouse and rat models of retinal degeneration.^142,143^ Viral delivery of rod-derived cone viability factor (RdCVF) is also under investigation for the treatment of RP; RdCVF is a factor naturally secreted by rods to protect cone photoreceptors,^154^ and has been found to promote photoreceptor survival in mouse models of RP after viral-mediated expression in the retina.^155^ If such strategies are effective in humans, this could be suitable for the significant number of IRD patients without a confirmed molecular diagnosis.

When choosing the method of treatment for Usher syndrome and other IRDs, the stage of disease will be an important consideration. The strategies already described are likely to be only effective at a stage where retinal photoreceptors are still intact. At the advanced stages of retinal degeneration, cell replacement therapies^156^ or retinal prosthesis^157^ may be the most feasible options. Advances in embryonic stem cell and iPSC technology make cell transplantation an ever-likely option for patients with late-stage retinal disease.^156^ Bone marrow–derived stem cells (BMSC) have been trialled in five ungenotyped patients with varying subtypes of Usher syndrome as part of the Stem Cell Ophthalmology Treatment Study (SCOTS; NCT01920867 and NCT03011541).^144^ Each Usher patient received autologous BMSC through either retrobulbar, subtenons, intravitreal, subretinal or intra-optic nerve injections into both eyes, followed by intravenous injections. The average pre-treatment logarithm of the minimum angle of resolution (LogMAR) acuity was 0.635, and the average postoperative change was a gain of 0.18 LogMAR. In the murine retina, it has been found that endogenous BMSC migrate and fuse with Müller glia cells after damage has been inflicted.^158^ The resulting hybrids were found to contribute to the replacement of damaged neurons, demonstrating the regenerative potential of BMSC in the mammalian retina.

Although there are no ongoing clinical trials for the Usher-specific hearing loss, there was a clinical trial for a recombinant adenovirus 5 (Ad5) vector containing the human atonal transcription factor (*ATOH1*) cDNA for administration via intra-labyrinthine infusion in patients with severe to profound sensorineural hearing loss (NCT02132130). The results are yet to be published. The successful preclinical work with several Usher mouse mutants makes gene therapy a promising future option; however, as discussed, these studies have involved treatment administration in prenatal or neonatal mice when the inner ear is still developing, and the use of similar therapies in children or adults with Usher syndrome may not be able to achieve reversal of the congenital inner ear defects.

## Conclusion

Usher syndrome is a disorder with vast clinical and genetic heterogeneity, typically resulting in significant dual sensory loss causing great impact on patient quality of life. More than ever, the prospect of an available treatment for at least some Usher subtypes looks promising. However, there are still obstacles to overcome in developing safe treatments that work for each gene size and mutation. In addition to the many gene- and mutation-specific treatments under investigation, finding universal treatments that use common mechanisms for the treatment of RP should be a priority for the many patients that remain without a molecular diagnosis. Further patient analysis is necessary to determine better genotype–phenotype correlations for each clinical subtype to predict prognosis; this will inform genetic counselling, preimplantation diagnosis and the choice of best outcomes for each treatment trial.
